# Morphological Phylogenetic Analysis of Seven Varieties of *Ficus deltoidea* Jack from the Malay Peninsula of Malaysia

**DOI:** 10.1371/journal.pone.0052441

**Published:** 2012-12-20

**Authors:** Hasan N. N. Fatihah, Nashriyah Mat, Abdul R. N. Zaimah, Mazlan N. Zuhailah, Haron Norhaslinda, Mahmud Khairil, Abdul Y. Ghani, Abdul M. Ali

**Affiliations:** 1 Department of Agricultural Science, Faculty of Agriculture and Biotechnology, Universiti Sultan Zainal Abidin, Gong Badak Campus, Terengganu, Malaysia; 2 Department of Biotechnology, Faculty of Agriculture and Biotechnology, Universiti Sultan Zainal Abidin, Gong Badak Campus, Terengganu, Malaysia; Kyushu Institute of Technology, Japan

## Abstract

This study is the first report to suggest a morphological phylogenetic framework for the seven varieties of *Ficus deltoidea* Jack (*Ficus*: Moraceae) from the Malay Peninsula of Malaysia. Several molecular-based classifications on the genus *Ficus* had been proposed, but neither had discussed the relationship between seven varieties of *F. deltoidea* to its allies nor within the varieties. The relationship between seven varieties of *F. deltoidea* is still debated due to the extreme morphological variabilities and ambiguous boundaries between taxa. Thus, the correct identification of these varieties is important as several morphological characters are variety-specific. To test the monophyly and further resolved the relationship in *F. deltoidea*, a morphological phylogenetic analysis was conducted based on herbarium specimens representing the seven varieties of *F. deltoidea* that were collected from the Malay Peninsula of Malaysia, by using related species of the genus *Ficus*; *F. grossularioides*, *F. ischnopoda* and *F. oleifolia* as the outgroups. Parsimony and neighbour-joining analyses indicated that *F. deltoidea* is monophyletic, in that the seven varieties of *F. deltoidea* nested into two clades; clade subspecies *deltoidea* (var. *deltoidea,* var. *bilobata,* var. *angustifolia,* var. *kunstleri* and var. *trengganuensis*) and clade subspecies *motleyana* (var. *intermedia* and var. *motleyana*).

## Introduction

*Ficus deltoidea* Jack (*Ficus*: Moraceae) is a diverse species of subgenus *Ficus*, section *Ficus* and subsection *Frutescentiae* [1]; which contains 25–30 species found in the Sino-Himalayan and western Malesian region [2]. *Ficus deltoidea* is a native and widely distributed throughout Malaysia, Thailand, Sumatra, Java, Kalimantan, Sulawesi and Moluccas [3]. This plant is a small shrub up to 3 m tall, sometimes occurring as an epiphyte [4]. It can be found in abundance along the beaches, peat soils and in hilly forest up to 3000 m above sea level [5]. The Malays called *F. deltoidea* as ‘*Mas Cotek’* due to the presence of golden dots on the upper surface of its lamina [5]. The seven varieties of *F. deltoidea;* namely var. *deltoidea,* var. *bilobata* Corner, var. *angustifolia* (Miq.) Corner, var. *intermedia* Corner, var. *kunstleri* (King) Corner, var. *motleyana* (Miq.) Corner and var. *trengganuensis* Corner that were found in the Malay Peninsula were described by Kochummen [6]. The plants are often recognized by the presence of golden dots on the upper surface of the lamina, dichotomous midrib, unique fig (syconia) with flowers hidden inside the syconia, leafy twigs and the milky juice [4], [7].

*Ficus deltoidea* is commonly cultivated as a houseplant for decorative purposes and traditional medicinal uses by the local people [4]. The var. *bilobata,* var. *angustifolia,* var. *intermedia,* var. *kunstleri,* var. *motleyana* and var. *trengganuensis* are commonly used in the Malay traditional medicine [8]. The dried leaves, stems and roots are often commercialized as herbal tea [9]. The decoction of the leaves is traditionally used by women after childbirth to help strengthen the uterus [5]. It is also believed to improve blood circulation, regain energy and enhance fertility naturally for both men and women [10], [11]. These claims were supported by previous bioassay studies, demonstrating that the aqueous extract of the leaves contains antidiabetic [12], [13] and antinociceptive activities [10]. The leaf extracts were reported to be rich of phenolic and flavonoid compounds which are comparable with black and green teas as well as fruit juices [11]. The flavan-3-ols and flavones were the main compounds that contributed to the total antioxidant activity [11], whereas isovetexin and vetexin were reported to be responsible for the antidiabetic activity [14].

Although *F. deltoidea* has been exploited in many different ways, the taxonomy of this species is still controversial at the varietal level. Historically, several botanical names of *F. deltoidea* have been reported; namely *F. diversifolia* Blume, *F. motleyana* Miq. and *F. oleifolia* King [4]–[15]. The extreme morphological variations and unclear boundaries between varieties create misleading identification of *F. deltoidea* varieties. The leaf characters are probably the most variable and showed heterophylly in the species [16]. Nevertheless, the young plant and the matured plant of the same variety often displayed different states of leaf characteristics. In this study, seven varieties collected from the Malay Peninsula of Malaysia, as mentioned by Kochummen [6], were selected, to investigate the monophyly of *F. deltoidea* and to differentiate intra-specific variation based on information contributed by overall morphological characters.

## Materials and Methods

### 1. Herbarium Specimens

Morphological data were scored from 108 herbarium specimens, with prior permissions from four different herbaria; the Herbarium of the Universiti Kebangsaan Malaysia (UKMB), Herbarium of the Forest Research Institute of Malaysia (FRIM), Herbarium of Sarawak (SAN) and the National Herbarium of Singapore (SING). All measurements and observations were taken from the herbarium specimens except data for flowers, which were gathered from the literatures [2], [4]–[7], due to limited number of syconium presents on each herbarium sample.

### 2. Specific Taxa Analyzed (Relevant Herbarium Specimens Examined are Listed in Alphabetical Order by Locality, Collector Names and Numbers)

*Ficus grossularioides* Burm. f. (Selangor: Anuar 115670 UKMB); *Ficus ischnopoda* Miq. (Kelantan: Zainudin 5726 UKMB); *Ficus oleifolia* King (Sarawak: Jamre 70886 SAN); *Ficus deltoidea* var. *angustifolia* (Miq.) Corner (Kelantan: Khairudin 31953 FRIM, Latiff 1042, 1785 UKMB, Whitmore 4186 SING; Terengganu: Burkill 804 FRIM, Latiff 2772 UKMB, Lim 54622 FRIM, Shah 3823 FRIM, Shah 3313 SING; Pahang: Henderson 21994 SING, Shah 2694 SING, Syed 23374 SING, Whitmore 4824 FRIM, Zainudin 2001 UKMB, Zainudin 5199 UKMB; Penang: Ogata 13358 FRIM, Ng 27351 FRIM; Perak: Borges 3404 SING, Chan 17503 FRIM, Henderson 10239 SING, Ridley 3036 SING, Ridley 10235 SING; Selangor: Henderson 10489 SING, Hume 9937 SING, Nur 34359 FRIM, Symington 18164 FRIM, SING); *F. deltoidea* var. *bilobata* Corner (Kelantan: Whitmore 4258 SING; Pahang: Chew 868 FRIM, Henderson 11077 SING, Latiff 3131 UKMB, Latiff 4085 UKMB, FRIM, Shah 2694 FRIM, Zainudin 4636 UKMB, FRIM; Perak: Nauen 1099 SING); *F. deltoidea* var. *deltoidea* Corner (Johor: Arishah 66 UKMB, Asiah 57 UKMB, Corner 32250 SING, Latiff 144, 698 UKMB, Mat 154 SING, Rahim 28 UKMB, Saidah 15 UKMB, Shafee 46 UKMB, Tan 102 FRIM, Teruya 434 SING, Zainal 20 UKMB; Kedah: Zainudin 3981 UKMB; Negeri Sembilan: Rasidah 023 UKMB, Satariah 52 UKMB; Pahang: Allen 111 SING, Zainudin 2336 UKMB); *F. deltoidea* var. *trengganuensis* Corner (Kelantan: Whitmore 4258 FRIM; Terengganu: Henderson 31039 FRIM, Kamarudin 219 UKMB, Kasim 1626 UKMB, Moysey 31075 FRIM, Rozi 67 UKMB, Sani 712 UKMB, Shah 4035 FRIM, Whitmore 12675 FRIM, Zainudin 3008 UKMB; Pahang: Ogata 10437 FRIM, Ridley 439 SING, Samsuri 464 FRIM, Yeoh 3112 SING). *F. deltoidea* var. *intermedia* Corner (Pahang: Asiah 086 UKMB, Asmah 72 UKMB, Henderson 23589 SING, Kasim 968 UKMB, Kiew 3823 SING, Littke 547 UKMB, Noraini 052 UKMB, Ridley 13717 SING, Rohani 068 UKMB, Saudah 056 UKMB, Shah 654 SING, Shah 2984 SING, Zainudin 1433 UKMB; Perak: Henderson 11818 SING). *F. deltoidea* var. *kunstleri* (King) Corner (Kelantan: Haniff 10230 SING, Henderson 29645 SING, Latiff 1041, 1899 UKMB; Pahang: Latiff 78 UKMB, Shah 1804 FRIM, Whitmore 3548 FRIM, Whitmore 3548 SING, Zainudin 17894 SING; Perak: Foston 45196 FRIM, Chelliah 98602 SING, Sanisah 1018 FRIM; Selangor: Mead 30761 FRIM); *F. deltoidea* var. *motleyana* (Miq.) C. C. Berg (Kelantan: Whitmore 20650 FRIM; Terengganu: Mahmud 4930 FRIM, Shah 4930 SING; Pahang: Ng 9726 FRIM, Symington 28908 SING, Whitmore 3227, 4848 FRIM).

### 3. Characters

The characters were surveyed throughout the ingroup and outgroup taxa using criteria of putative homology or hypothetical homology [17]. Furthermore, the characters should consistent in occurrence or absence among the terminal taxa, which implies that they are not environmentally plastic [18]. As a basic principle, the characters that varied between terminal taxa were chosen, but not those that varied within the taxa. In total, 32 morphological characters including 29 binary and three multistate characters were prepared (Table 1). Character states were then polarized using outgroup comparison method [19]. If a character state was not available or not applicable in a taxa, it was designated as missing.

**Table 1:** List of morphological characters and character states used in the phylogenetic analysis.

No.	Characters	Character states
1	Leaf length	0: more than 5 cm, 1: equal or less than 5 cm.
2	Leaf width	0: more than 5 cm, 1: equal or less than 5 cm.
3	Midrib	0: not forked to forked near the apex, 1: forked near the middle of the lamina.
4	Angle of the forked midrib	0: not forked or forked less than 45 degrees, 1: forked more than 45 degrees.
5	Leaf apex	0: acute to acuminate, 1: rounded to truncate and minutely emarginate to form bilobed.
6	Leaf base	0: obtuse, 1: acute.
7	Leaf shape	0: oblanceolate, 1: obovate, 2: spatulate.
8	Leaf margin when dried	0: serrate, 1: entire, 2: wavy.
9	Leaf surface	0: veins deeply impressed, 1: plane or veins slightly impressed.
10	Leaf venation	0: open venation, 1: close venation.
11	Waxy gland beneath the lamina	0: two, 1: equal or more than three.
12	Gland at the forked midrib	0: absent, 1: present.
13	Gland at the subsequent dichotomies of the midrib	0: absent or rarely seen, 1: commonly seen.
14	Petiole length	0: more than 1.5 cm, 1: equal or less than 1.5 cm.
15	Petiole indument at tip	0: puberulous, 1: glabrous.
16	Stipule length	0: more than 0.5 cm, 1: equal or less than 0.5 cm.
17	Periderm persistent	0: present, 1: absent.
18	Peduncle length	0: equal or less than 1 cm, 1: more than 1 cm.
19	Fig type	0: in pairs, 1: solitary.
20	Fig indumenta	0: puberulous, 1: glabrous.
21	Fig shape	0: globose, 1: oblong.
22	Fig length	0: equal or less than 1 cm, 1: more than 1 cm.
23	Fig width	0: more than 0.5 cm, 1: equal or less than 0.5 cm.
24	Fig base	0: cupulate, 1: cuneate.
25	Fig apex	0: concave, 1: protracted.
26	Ratio of fig length/width	0: more than 1, 1: less than 1.
27	Ostiole diameter	0∶1.5–2.5 mm, 1: less than 1.5 mm.
28	Color of ripening figs	0: yellow to orange to brownish red, 1: rose red to dark purple.
29	Tepals long over ovary	0: shorter than ovary, 1: as long as ovary, 2: longer than ovary.
30	Tepals color	0: red to dark purple, 1: yellowish red.
31	Ovary shape	0: rugose-angular, 1: smooth or slightly angular.
32	Ovary exocarp	0: crustaceous, 1: fleshy.

### 4. Phylogenetic Analyses

The phylogenetic analyses were performed using the maximum parsimony (MP) and Wagner approaches and trees were generated using the PAUP* version 4.0b10 software [20], [21]. All character states were run as unordered with equal weight. The search for the most parsimonious tree was determined by exhaustive search and bisection-reconnection (TBR) branch swapping, with retention of multiple parsimonious trees (MaxTrees = 100). Branches were collapsed and polytomies were created when maximum branch length is zero. Optimization of characters was performed using the ACCTRAN (Accelerated Transformation Optimization) option. To test the support for each clade, bootstrap analysis [21] was performed with 1000 replicates of simple taxon addition and TBR swapping, with a limit of 10 trees kept per replicate. Bootstrap percentages (BS) of 50–70 was considered weak, 71–85 as moderate and >85 as strong [22]. The distance tree was estimated by the neighbour-joining (NJ) method [23] based on the formulae of Kimura [24]. Graphic outputs were produced using the TreeView X software [25] and characters were mapped onto a single tree [26].

## Results

**Figure 1:**
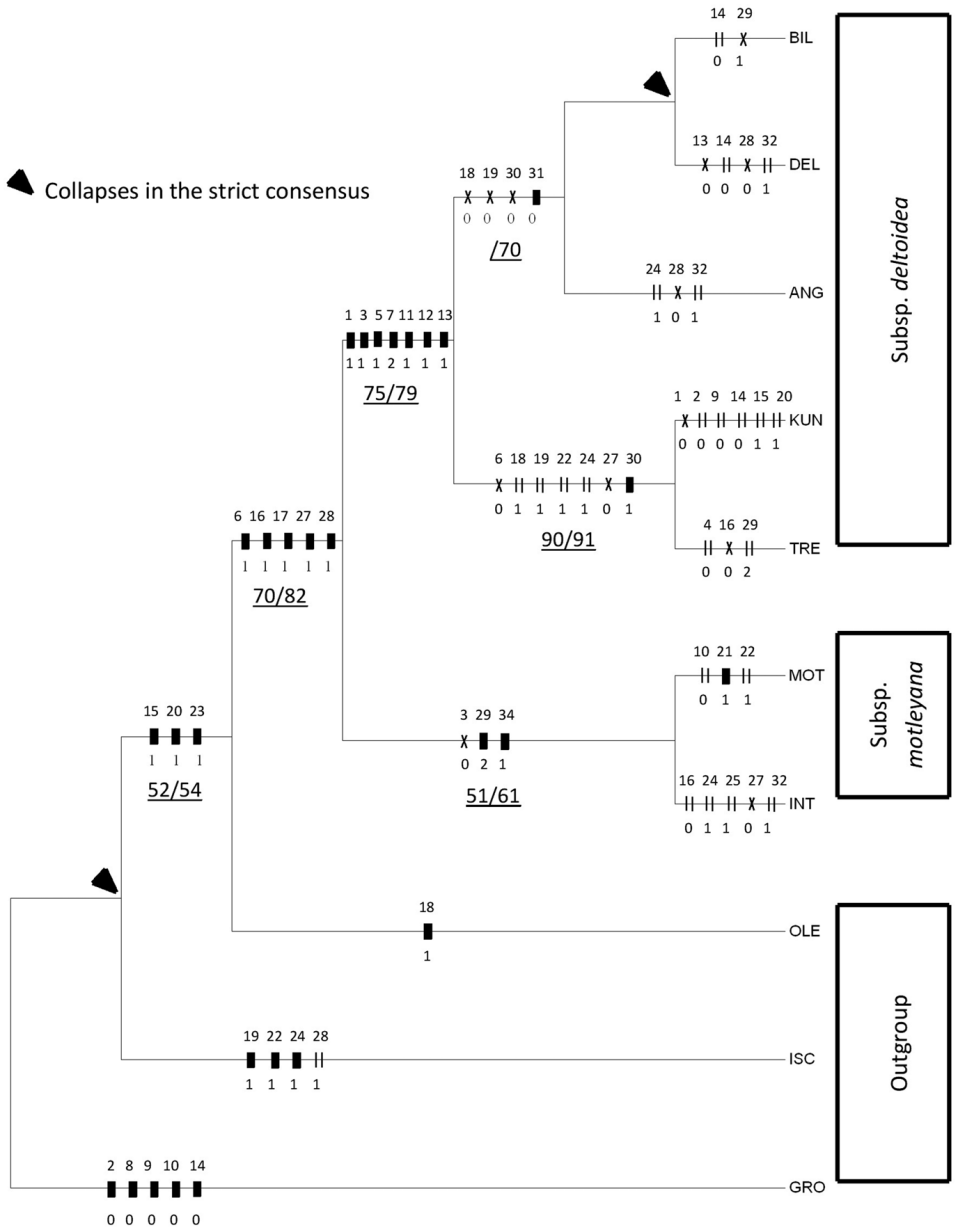
Neighbour-joining tree of *F. deltoidea* resulting from the morphological data. Terminal taxa: GRO = *F. grossularioides*, ISC = *F. ischnopoda*, OLE = *F. oleifolia,* DEL = var. *deltoidea*, BIL = var. *bilobata*, ANG = var. *angustifolia*, KUN = var. *kunstleri*, TRE = var. *trengganuensis*, MOT = var. *motleyana*, INT = var. *intermedia*. Bars = synaphomorphies; parallel lines = parallelisms; crosses = reversals. Underlined numbers below the branches are bootstrap percentage value of maximum parsimony/neighbour-joining analyses.

The resultant data matrix is shown in Table S1. The analysis of the data matrix, containing nine terminal taxa and 32 characters, produced two shortest maximum parsimony (MP) trees with a minimum length of 68 steps, a consistency index (CI) of 0.5147 and a retention index (RI) of 0.5976. A total of 31 parsimony-informative characters and only one parsimony-uninformative character were found in the dataset. The only difference between the two trees topologies is the position of var. *motleyana* and var. *intermedia,* which was supported and characterized by tepals longer than ovary (#29) and a synapomorphy of smooth and slightly angular ovary (#31). There was no character found to support this group, thus collapsed into polytomy in the other tree. Other character transformation series within the whole tree were found to be almost identical in both trees. The results were then compared with the neighbour-joining (NJ) tree (Figure 1). Noted that the topology was similar in MP and NJ trees, but the bootstrap supports (BS) of NJ tree were generally improved compared to the MP tree.

*Ficus deltoidea* formed a weakly-moderately supported clade with bootstrap support of 70% and 82% in the MP and NJ trees, respectively. The group was characterized by the important characters of leaf base acute (#6), stipule length equal or less than 0.5 cm (#16), periderm not persistent (#17), ostiole diameter less than 1.5 mm (#27) and color of ripening fig that is rose red to dark purple (#28). The species was divided into two clades; clade subspecies *deltoidea* (BS  = 75%/79%) and clade subspecies *motleyana* (BS  = 51%/61%), of which the first clade comprised of five varieties namely, var. *angustifolia,* var. *bilobata,* var. *deltoidea,* var. *kunsleri* and var. *trengganuensis,* whereas the second clade contained two varieties namely, var. *intermedia* and var. *motleyana.*

The placement of clade subspecies *deltoidea* was moderately supported with 75% and 79% bootstrap values in the MP and NJ analyses, respectively. It was defined by the following characters; leaf length equal or less than 5 cm (#1), midrib forked near the middle of the lamina (#3), leaf apex rounded to truncate and minutely emarginate to form bilobed (#5), leaf spatulate (#7), waxy gland beneath the lamina is equal or more than three (#11), gland present at the forked midrib (#12) and commonly seen at the subsequent dichotomies of the midrib (#13). Within subspecies *deltoidea*, var. *kunsleri* and var. *trengganuensis* formed a strongly supported clade, 90% and 91% in the MP and NJ trees, respectively. They were described by having obtuse leaf base (#6), peduncle length more than 1 cm (#18), fig borne in pairs (#19), fig length more than 1 cm (#22), fig cuneate at the base (#24), ostiole diameter between 1.5–2.5 mm (#27) and flowers with yellowish-red tepals (#30). However, var. *kunstleri* was discriminated from its ally by having leaf length and width that is more than 5 cm each (#1), (#2), veins are deeply impressed (#9), petiole length more than 1.5 cm (#14), glabrous at the tip (#15) and glabrous fig (#20), whereas var. *trengganuensis* was identified by having midrib forked less than 45 degrees (#4), stipule length more than 0.5 cm (#16) and tepals longer than ovary (#29). The positions of var. *deltoidea*, var. *bilobata* and var. *angustifolia* were defined by extremely low confidence in the MP analysis and therefore, relationship between these varieties were not certain. However, the NJ analysis support their relationship by 70% bootstrap support and they share the peduncle length that is equal or less than 1 cm (#18), fig solitary (#19), flowers with red and dark purple tepals (#30) and rugose-angular ovary (#31). Within this group, var. *angustifolia* was identified by having fig cuneate at the base (#24), ripening fig yellow to orange to brownish red (#28) and ovary with fleshy exocarp (#32), whilst var. *bilobata* showed a unique autapomorphy of tepals length that is as long as ovary (#29).

The clade subspecies *motleyana* received weak bootstrap support of 51% in the MP analysis, but was moderately supported, 61% in the NJ analysis. It was distant to subspecies *deltoidea* by having midrib not forked to forked near the apex (#3), tepals longer than ovary (#29) and smooth or slightly angular ovary (#31). Within subspecies *motleyana,* var. *intermedia* was distinguished by having stipule length more than 0.5 cm (#16), fig cuneate at the base (#24), protracted at the apex (#25), ostiole diameter between 1.5–2.5 mm (#27) and ovary with fleshy exocarp (#32). On the other hand, var. *motleyana* showed an autapomorphy of fig oblong in shape (#21).

## Discussion

Most of the previous classifications (Table 2) were based on intuitive morphology. The number of varieties was easily increased or reduced based on its morphological variation and locality [27]. Different authors had their own opinion in discriminating taxon, such as Corner [28] who divided the South East Asian species of *F. deltoidea* into twelve varieties and four forma, namely, var. *deltoidea,* var. *angustifolia* f. *angustissima* Corner, var. *arenaria* Corner, var. *bilobata*, var. *borneensis* Corner f. *subhirsuta* Corner, var. *intermedia*, var. *kunstleri*, var. *lutescens* (Desf.) Corner f. *longipedunculata* Corner, f. *subsessilis* (Miq.) Corner, var. *motleyana*, var. *oligoneura* (Miq.) Corner, var. *peltata* Corner and var. *trengganuensis*. Later, he introduced a new variety, var. *kinabaluensis* Stapf which probably is a synonym of var. *intermedia* of Borneo with larger peduncle and leaves [4]. On the other hand, Kochummen [4] has divided *F. deltoidea* into seven varieties namely, var. *deltoidea,* var. *bilobata,* var. *angustifolia,* var. *intermedia,* var. *kunstleri,* var. *motleyana* and var. *trengganuensis*, which were available in the Malay Peninsula of Malaysia or formerly known as Malaya, and described them. Later on, two endemic varieties of Borneo, namely var. *subhirsuta* Kochummen and var. *recurvata* Kochummen were added [27]. Berg [2] and Berg and Corner [7] recently subdivided the species into two major morphological entities (subspecies *deltoidea* and subspecies *motleyana*) which seems to be more practical and satisfactory in handling the variation. They further mentioned that as strong phytogeographical and ecological support is lacking, the rank of variety proposed by others might be more correct, but the chosen rank allows recognition of varieties for regional use. Therefore, the sampling of this study focused on the earlier classification scheme of Kochummen [6] who studied seven varieties from Peninsular Malaysia region. These varietal variations then might be useful for allocating plant material to varieties as required for therapeutic and pharmaceutical applications.

**Table 2:** Fluctuation of taxonomic rank in *F. deltoidea* based on geographic regions.

Author	Corner (1960)	Corner (1969)	Kochummen (1978)	Kochummen (1998)	Kamarudin and Latiff (2002)	Berg (2003); Berg and Corner (2005)
**Geographic Regions**	South East Asia	South East Asia	Peninsular Malaysia	Malaysia and Borneo	Malaysia	Malesia
	–	–	–	–	–	**A) Subspecies ** ***deltoidea***
	v. *angustifolia*	v. *angustifolia*	v. *angustifolia*	v. *angustifolia*	v. *angustifolia*	v. *angustifolia*
	f. *angustissima*	f. *angustissima*	–	–	–	f. *angustissima*
	v. *arenaria*	v. *arenaria*	–	–	–	v. *arenaria*
	v. *bilobata*	v. *bilobata*	v. *bilobata*	v. *bilobata*	v. *bilobata*	v. *bilobata*
	v. *borneensis*	v. *borneensis*	–	–	–	v. *borneensis*
	f. *subhirsuta*	f. *subhirsuta*	–	v. *subhirsuta*	–	f. *subhirsuta*
	v. *intermedia*	v. *intermedia*	v. *intermedia*	v. *intermedia*	v. *intermedia*	
**Taxa**	v. *kunstleri*	v. *kunstleri*	v. *kunstleri*	v. *kunstleri*	v. *kunstleri*	v. *kunstleri*
	v. *lutescens*	v. *lutescens*	–	–	–	v. *lutescens*
	f. *longipedunculata*	f. *longipedunculata*	–	–	–	f. *longipedunculata*
	f. *subsessilis*	f. *subsessilis*	–	–	–	f. *subsessilis*
	v. *peltata*	v. *peltata*	–	–	–	v. *peltata*
	v. *trengganuensis*	v. *trengganuensis*	v. *trengganuensis*	v. *trengganuensis*	v. *trengganuensis*	v. *trengganuensis*
	v. *deltoidea*	v. *deltoidea*	v. *deltoidea*	v. *deltoidea*	–	
	–	v. *kinabaluensis*	–	–	–	
	–	–	–	v. *recurvata*	–	v. *recurvata*
	–	–	–	–	–	**B) Subspecies ** ***motleyana***
	v. *motleyana*	v. *motleyana*	v. *motleyana*	v. *motleyana*	v. *motleyana*	v. *motleyana*
	v. *oligoneura*	v. *oligoneura*	–	–	–	v. *oligoneura*

“v.” is referred to variety and “f.” is forma.

“–“ means not found and not described.

In this study, *Ficus deltoidea* formed one major clade. Nevertheless, character variation in the dataset was insufficient to resolve all the phylogenetic relationships, especially among the internal branching of subspecies *deltoidea*. The divergence of var. *deltoidea,* var. *angustifolia* and var. *bilobata* received extremely low bootstrap support, thus the branches collapsed in the strict consensus tree. The placement of these varieties, as revealed by a previously published RAPD analysis, were also uncertain due to the variable banding patterns and hence showed that similar varieties were grouped into different clusters [9].

From the results obtained, the seven varieties of *F. deltoidea* of Malesian region can be placed into two subspecies: subspecies *deltoidea* consisted of var. *deltoidea,* var. *bilobata,* var. *angustifolia,* var. *kunstleri* and var. *trengganuensis*. Within the subspecies *deltoidea*, var. *kunstleri* was shown to be more closely related to var. *trengganuensis* than to the other varieties. The positions of var. *deltoidea*, var. *bilobata* and var. *angustifolia* received extremely low support in the MP tree, suggesting that the analysis did not assure the relationships between these varieties. However, they were genetically closely related to each other and were fairly supported in the NJ analysis. This should, however, be confirmed by a detailed molecular studies. The second, subspecies *motleyana* comprised var. *intermedia* and var. *motleyana*. Our results showed an agreement with Berg [2], and Berg and Corner [7] that proposed the two subspecies based on the forked and non-forked midrib. Noted that var. *intermedia* was excluded in their classifications and then was transferred to *F. oleifolia* subspecies *intermedia,* because of mixture in characters of forked and non-forked midrib, and leaf shapes ranging from spatulate to obovate to oblanceolate [2], [7]. However, this study showed that the relationship between var. *intermedia* and var. *motleyana* was constantly supported and defined in both the MP and NJ analyses.

With respect to intergeneric relationship between *F. deltoidea, F. ischnopoda* Miq., *F. oleifolia* King and *F. grossularioides* Burm.f., *F. oleifolia* was found to be the closest ally to *F. deltoidea*, whilst *F. ischnopoda* and *F. grossularioides* were placed at the base of trees and did not get any bootstrap support (Figure 1). Their relationships, however, continue to require explicit examination using a combination of molecular and developmental dataset. It is interesting to note that Rønsted *et al.,* [29] groups *F. deltoidea* var. *borneensis* and *F. oleifolia* together with *F. adenosperma* Miq., *F. ochrochlora* Ridl., *F. dammaropsis* Diels, *F. pumila* L., *F. erecta* Thunb., and placed *F. ischnopoda* at the base of section *Ficus/Adenosperma* on the basis of several molecular datasets. Furthermore, Rønsted *et al.,* [30] recently found that *F. oleifolia* and *F. ischnopoda* were the closer relatives to *F. deltoidea* var. *borneensis* in subsection *Frutescentiae*, whilst *F. grossularioides* more remotely in section *Eriosycea*. The sampling of subsection *Frutescentiae* and *F. deltoidea* varieties has been very limited in the previous phylogenetic studies, which makes it difficult to know what would be the close relatives and appropriate outgroup. Therefore, it is not possible at this stage to compared *F. deltoidea* to its allies because there is no comprehensive phylogenetic classification available for comparison.

This study was the first attempt to suggest a morphological phylogenetic framework for the seven varieties of *Ficus deltoidea* from the Malay Peninsular, which will provide a basis for future molecular, cytological or phytochemical as well as pharmaceutical investigations. Deeper understanding of the systematic relationships between the varieties will help to promote expeditious exploitation and sustainable uses of this plant as a whole.

## Supporting Information

Table S1 Data matrix for 32 morphological characters. “0” represents the plesiomorphic state, and “1” or “2” represents the apormorphic state. Missing data are indicated by “?”.
(DOC)
