# Correction: Morphological Phylogenetic Analysis of Seven Varieties of *Ficus deltoidea* Jack from the Malay Peninsula of Malaysia

**DOI:** 10.1371/annotation/92e7b830-9db4-47cf-9851-8fa939b1dc2c

**Published:** 2013-08-06

**Authors:** Hasan N. N. Fatihah, Mat Nashriyah, Abdul R. N. Zaimah, Mazlan N. Zuhailah, Haron Norhaslinda, Mahmud Khairil, Abdul Y. Ghani, Abdul M. Ali

The name of the second author was presented incorrectly in the citation.

The correct citation is: Fatihah HNN, Nashriyah M, Zaimah ARN, Zuhailah MN, Norhaslinda H, et al. (2012) Morphological Phylogenetic Analysis of Seven Varieties of *Ficus deltoidea* Jack from the Malay Peninsula of Malaysia. PLoS ONE 7(12): e52441. doi:10.1371/journal.pone.0052441 

